# New fixed points of mixed monotone operators with applications to nonlinear integral equations

**DOI:** 10.1371/journal.pone.0325762

**Published:** 2025-06-13

**Authors:** Shaoyuan Xu, Yan Han, Shixun Lin, Guoqiong Zhou

**Affiliations:** School of Mathematics and Statistics, Zhaotong University, Zhaotong, Yunnan, China; Northwestern Polytechnical University, CHINA

## Abstract

In this paper, first we recall and present some basic results about cone and partial order within the framework of Banach spaces. Next, by means of the properties of cone and monotone iterative techniques, some new fixed point theorems of mixed monotone operators with certain concavity and convexity are obtained without any compactness or continuity condition. Further, the main results are applied to two classes of nonlinear functional integral equations on unbounded regions. Our results extend and generalize previous findings.

## 1 Introduction and preliminaries

Since the notion of mixed monotone operator was established in 1987 (see [1]), a number of scholars have explored the existence and uniqueness of different types of fixed points for such operators (see [1–18]-20]). In order to solve the fixed point problems, two common methods are usually utilized in the study of mixed monotone operators. One is to use the compactness or continuity of the operators whenever such condition is satisfied (see [1–4]), the other is to take the advantage of certain concavity or convexity by means of cone theory and monotone iterative techniques once such concavity or convexity property lies in the operators (see [5–21]). These methods have been widely applied in various fields, including integral equations (see [22, 23]), higher-order mixed integro-differential equations (see [24–33]), fractional integro- differential equations (see [34–37]) and some other related applications (see [38–44]). These works demonstrate the versatility and applicability of mixed monotone operators in various mathematical problems.

Among them, the authors in [11] presented a new concept, named ϕ concave-(−ψ) convex mixed monotone operators. They gained several theorems of this type of operators with certain concavity and convexity. The advantage of introducing ϕ concave-(−ψ) convex mixed monotone operators is that it can unify a large number of mixed monotone operators with certain concave-convex properties, and thus cope with the fixed point problems together in a general way. However, in the existing literature, such as [11], a crucial question arises about the fixed point theorem of ϕ concave-(−ψ) convex operators, which is as following:

Question A: If we delete the condition (C) :

(C) $\lim_{s\to t^{-}}\phi (s,w_{0})\psi (s,w_{0})>t$

in Theorem 2.1 in [11], then do the fixed point results in Theorem 2.1 in [11] remain true?

In this paper, we positively answer this question. Following [11], we continue to investigate the fixed point problems regarding ϕ concave-(−ψ) convex mixed monotone operators. Without requiring any compactness or continuity of the operators, we also get the existence and uniqueness conclusions for these operators, as well as the convergence of the iterative sequences. The novelty of this work is that we utilize a new iterative technique to establish some new fixed point theorems of mixed monotone operators in which the existence and uniqueness of the fixed points, as well as the convergence of iterative sequences are proven by only requiring weaker conditions than that in [11] and [20], thus improve the main results in [11] and [20] greatly. Furthermore, we also obtain a number of new fixed point results about mixed monotone operators with certain concavity and convexity without requiring coupled upper or lower solutions. In addition, the main results are applied to two types of nonlinear integral equations. Our work extends and improves the previous related works.

Now we review some basic definitions, notations and known facts in the theory of cone and partial order, which can be found in [1, 19].

A real Banach space *S* is commonly defined as a real normed vector space where all Cauchy sequences must converge in *S*.

Let *S* be a real Banach space and *U* be a subset of *S*. Denote by θ the null element of *S* and by int*U* the interior of *U*. The subset *U* is called a cone if:

(i) ϖ∈U and λ≥0, then λϖ∈U

(ii) λU⊂U(∀λ≥0), i.e., U∩(−U)={θ}, i.e., ϖ∈U and −ϖ∈U, then ϖ=θ.

**Remark 1.1**   Let the cone U⊂S. Then we have U+U⊂U since for any σ=ϖ+ϱ∈U+U (where ϖ,ϱ∈U), $\sigma =\varpi +\varrho =2(\frac{1}{2}\varpi +\frac{1}{2}\varrho )\in U$.

Given a cone U⊂S, we define a partial ordering ≤ with respect to *U* by ϖ≤ϱ if and only if ϱ−ϖ∈U. We shall write ϖ<ϱ if ϖ≤ϱ and ϖ≠ϱ, while ϖ≪ϱ will stand for ϱ−ϖ∈intU when intU≠∅. A cone *U* is called normal if there exists $k\in {\mathbb{R}}^{+}$ such that for all ϖ,ϱ∈U,


θ≤ϖ≤ϱimplies∥ϖ∥≤k∥ϱ∥.

The least positive number of *k* is said to be the normal constant of *U*.

A cone *U* is called solid, if intU is not empty, i.e., intU≠∅.

Let M⊂S. An operator ℱ:M×M→S is said to be mixed monotone if ℱ(ϖ,ϱ) is nondecreasing in ϖ and nonincreasing in ϱ, i.e., $\forall \varpi_{i},\varrho_{i}\in M\;(i=1,2),\varpi_{1}\leq \varpi_{2}$ and $\varrho_{2}\leq \varrho_{1}$ imply $ℱ(\varpi_{1},\varrho_{1})\leq ℱ(\varpi_{2},\varrho_{2})$. An element $\varpi^{*}\in M$ is named a fixed point of ℱ if $ℱ(\varpi^{*},\varpi^{*})=\varpi^{*}.$ ℱ:M⊂S→S is said to be convex if for ϖ,ϱ∈M with ϖ≤ϱ and each t∈[0,1] we have


ℱ(tϖ+(1−t)ϱ)≤tℱϖ+(1−t)ℱϱ;

ℱ is said to be concave if −ℱ is convex.

Let e>θ, write


$$U_{e}=\{\varpi \in S\;|\text{\textasciitilde{}there exist}\;\lambda ,\mu >0,\text{\textasciitilde{}such that}\;\lambda e\leq \varpi \leq \mu e\}.$$

If ℱϖ=ϖ for some ϖ∈M, then ϖ is named a fixed point of ℱ in *M*. The operator ℱ:M→S is said to be increasing or nondecreasing, if for any ϖ,ϱ∈M , ϖ≤ϱ implies ℱϖ≤ℱϱ.

Let $\iota_{0},\kappa_{0}\in S$ and $\iota_{0}\leq \kappa_{0}$, then


$$\iota_{0}\leq \kappa_{0}$$

is said to be an ordering interval.

**Definition 1.1** ([11])  Let *M* = *U* or M=intU and α∈ℝ with 0≤α<1. Let ℱ:M→M be an operator.

(1) ℱ is said to be α-concave, if for any ϖ∈M and 0<t<1, $ℱ(t\varpi )\geq t^{\alpha }ℱ\varpi$;

(2) ℱ is said to be (−α)-convex, if for any ϖ∈M and 0<t<1, $ℱ(t\varpi )\leq t^{-\alpha }ℱ\varpi$.

**Remark 1.2** ([11])  Any α-convex operator (α>1) must be an *e*-convex operator, where the characteristic function $\eta (t,\varpi )=1-t^{\alpha -1}$.

**Definition 1.3** ([11])  Let M⊂S. An operator ℱ:M×M→S is said to be ϕ concave-(−ψ) convex, if there exists a function ϕ:(0,1]×M→(0,∞) and a function ψ:(0,1]×M→(0,∞) such that (t,ϖ)∈(0,1)×M implies t<ϕ(t,ϖ)ψ(t,ϖ)≤1 and also ℱ satisfies the following two conditions:

$(H_{1})\;ℱ(t\varpi ,\varrho )\geq \phi (t,\varpi )ℱ(\varpi ,\varrho ),\forall t\in (0,1),\forall (\varpi ,\varrho )\in M\times M$;

$(H_{2})\;\;ℱ(\varpi ,t\varrho )\leq \frac{1}{\psi (t,\varrho )}ℱ(\varpi ,\varrho ),\forall t\in (0,1),\forall (\varpi ,\varrho )\in M\times M$.

**Definition 1.4** ([20])  Let ℱ:U→U be an operator and e>θ. Suppose that

(i)  $ℱe\in U_{e}$,

(ii)  there is a real number η=η(t,ϖ)>0 satisfying

$$ℱ(t\varpi )\geq (1+\eta )tℱ\varpi ,\forall \varpi \in U_{e},\;\forall t\in (0,1).$$ (1)

Then ℱ is said to be a generalized *e*-concave operator, and η=η(t,ϖ) is named its characteristic function.

Similarly, in the above-mentioned definition, if the condition (ii) is replaced by the following

(ii’) $ℱ(t\varpi )\leq \frac{1}{t(1+\xi )}ℱ\varpi ,\forall \varpi \in U_{e},\;\forall t\in (0,1),$

then ℱ is called a generalized *e*-convex operator, and ξ=ξ(t,ϖ) is called its characteristic function.

## 2 Some basic results on abstract cones in order real Banach spaces

In this section, we will show some useful and interesting results about abstract cone and partial order in real ordered Banach spaces.

In what follows, the set *S* always represents a real Banach space.

**Lemma 2.1**  Assume the cone U⊂S is solid and e∈intU. Then for any ϖ∈S, there exists a sufficient small number λ>0 such that e−λϖ∈U.

**Proof.**  For any ϖ∈S, without loss of generality, assume that ϖ≠θ. Since e∈intU, it follows that there is a sufficient small number *r*>0 such that

{ϖ∈S:‖ϖ−e‖≤r}⊂U. (2)

Obviously, we see

$$\left\|e-\frac{r}{\left\|\varpi \right\|}\varpi -e\right\|=\left\|\frac{-r}{\left\|\varpi \right\|}\cdot \varpi \right\|=r\leq r.$$ (3)

By 2.1 and 2.2 we have $e-\frac{r\varpi }{\left\|\varpi \right\|}\in U$. Set $\lambda =\frac{r}{\left\|\varpi \right\|}>0$, then there exists sufficient small λ>0 such that e−λϖ∈U, which completes the proof of Lemma 2.1.

By Lemma 2.1, we can deduce a number of useful results about cone and partial order.

**Corollary 2.1**  Assume the cone U⊂S is solid and ϖ,ϱ∈S. If ϖ≫ϱ, then there exists a real number η>0 such that ϖ≥(1+η)ϱ.

**Proof.**  Since ϖ≫ϱ, set e=ϖ−ϱ, then e∈intU. By Lemma 2.1, it follows that for any σ∈S, there exists a real number λ>0 such that


ϖ−ϱ−λσ∈U.

Take σ=ϱ,η=λ, then we have


ϖ−ϱ−ηϱ≥θ,

which implies ϖ≥(1+η)ϱ, a desired result.

**Remark 2.1**  In Corollary 2.1 above, we see the conclusion ϖ≥(1+η)ϱ implies ϱ≤εϖ, where $\varepsilon =\frac{1}{1+\eta }$ and so 0<ε<1. Hence Corollary 2.1 can deduce [45], Lemma 3.1], that is to say, Corollary 2.1 generalizes [45], Lemma 3.1].

**Proposition 2.1** ([46])  Assume the cone U⊂S and ϖ,ϱ,σ∈S.

(i) ϖ≥ϱ,ϱ>σ⇒ϖ>σ;

(ii) ϖ>ϱ,ϱ≥σ⇒ϖ>σ.

Furthermore, suppose *U* is solid, we have

(iii) intU⊂U, so ϖ≫ϱ⇒ϖ≥ϱ;

(iv) ϖ≫ϱ,ϱ≥σ⇒ϖ≫σ;

(v) ϖ≥ϱ,ϱ≫σ⇒ϖ≫σ;

(vi) λ>0⇒λintU⊂U;

(vii) ϖ≫θ,λ>0⇒λϖ≫θ; ϖ≫ϱ,λ>0⇒λϖ≫λϱ.

**Proof.**  (i) Suppose ϖ≥ϱ,ϱ>σ, then ϱ≥σ, so we see ϖ−ϱ∈U and ϱ−σ∈U. Hence ϖ−σ=(ϖ−ϱ)+(ϱ−σ)∈U since U+U⊂U. That is, ϖ≥σ. Now let us show ϖ≠σ. Otherwise if ϖ=σ, then by ϖ≥ϱ we see σ≥ϱ, i.e., σ−ϱ∈U. On the other hand, since ϱ≥σ, i.e., ϱ−σ∈U, we have σ−ϱ∈(−U). So σ−ϱ∈U∩(−U)={θ}, which implies σ−ϱ=θ, i.e., ϱ=σ, a contradiction to ϱ>σ. Thus ϖ≠σ. Therefore, ϖ>σ.

(ii) similar to (i).

(iii) Suppose e∈intU, by Lemma 2.1, for any ϖ∈S, there exists λ>0 such that e−λϖ∈U. Taking ϖ=θ, then we get e=e−λϖ∈U. Hence, intU⊂U. Besides, if ϖ≫ϱ, then ϖ−ϱ∈intU, so by the arguments above we have ϖ−ϱ∈U, i.e., ϖ≥ϱ.

(iv) Suppose ϖ≫ϱ,ϱ≥σ. Noting that a basic fact as follows: for any ς∈S and *r*>0, the ball B(ς,r) in *S* can be written as


B(ς,r)=ς+B(θ,r),

where B(ς,r)={ϱ∈S|‖ϱ−ς‖<r}. Then we see ϖ≫ϱ⇒ϖ−ϱ∈intU⇒ there exists *r*>0 such that


ϖ−ϱ+B(θ,r)=B(ϖ−ϱ,r)⊂U,

hence

ϖ−ϱ+B(θ,r)⊂U. (4)

Since ϱ≥σ, it follows that

ϱ−σ∈U. (5)

By 2.3 and 2.4 we have


ϖ−σ+B(θ,r)=ϱ−σ+ϖ−ϱ+B(θ,r)⊂U+U⊂U,

which implies B(ϖ−σ,r)⊂U. That is, ϖ−σ∈intU. So, ϖ≫σ.

(v) Similar to (iv).

(vi) Suppose λ>0,ϖ∈intU, we need to prove λϖ∈intU, i.e., there exists *r*_1_>0 such that

$$B(\lambda \varpi ,r_{1})\subset U.$$ (6)

Since ϖ∈intU, there exists *r*>0 such that


ϖ+B(θ,r)=B(ϖ,r)⊂U.

So, for any ϱ∈B(θ,r), we get ϖ+ϱ∈U. Hence, it follows from λU⊂U(λ>0) that

λϖ+λϱ∈λU⊂U, (7)

So, λϖ+λϱ∈λU. Now, we prove

B(λϖ,λr)⊂U, (8)

i.e.,


λϖ+B(θ,λr)⊂U.

In fact, for any σ∈B(θ,λr), we have ‖σ‖≤λr. In order to prove 2.7, it suffices to show λϖ+σ∈U. Indeed, setting $\varrho =\frac{1}{\lambda }\sigma$, then we see $\|\varrho \|=\frac{1}{\lambda }\|\sigma \|<\frac{1}{\lambda }\cdot \lambda r=r$, i.e., ϱ∈B(θ,r). So by 2.6 we have λϖ+λϱ∈U, i.e., λϖ+σ∈U. Thus, 2.7 holds, which implies 2.5 holds by taking $r_{1}=\lambda r$.

(vii) It is obvious that (vi) implies (vii). We only prove the second conclusion in (vii), namely, ϖ≫ϱ,λ>0⇒λϖ≫λϱ. The proof of the first conclusion in (vii) is obviously seen when one takes ϱ=θ in the second one in (vii). Set σ=ϖ−ϱ, then ϖ≫ϱ implies σ≫θ, i.e., σ∈intU. So by (vi) we have


λσ∈λintU⊂intU

since λ>0. Thus × i.e., ×. So ×.

**Lemma 2.2**  Assume the cone U⊂S is solid and ϖ∈S. Then ϖ∈intU if and only if there is a point e∈intU and a real number λ>0 such that ϖ≥λe.

**Proof.**  (⇒): Assume ϖ∈intU. Taking e=ϖ,λ=1, then we see ϖ=1·ϖ≥λe. So ϖ≥λe.

(⇐): Assume there is a point e∈intU and a real number λ>0 such that ϖ≥λe. Then by Proposition 2.1 (vii) we see $\left\{ \begin{array}{c} L_{\text{WIoUv1}}=R_{\text{WIoU}}L_{\text{IoU}}\\ R_{\text{WIoU}}=\exp\left(\frac{(x-x_{gt}{)}^{2}+(y-y_{gt}{)}^{2}}{(W_{g}^{2}+H_{g}^{2}{)}^{*}}\right) \end{array}\right.$ and $\left\{ \begin{array}{c} \beta =\frac{L_{IoU}^{*}}{L_{IoU}^{**}}\in [0,+\infty )\\ L_{WIoUv3}=rL_{WIoUv1},\;r=\frac{\beta }{\delta \alpha^{\beta -\theta }} \end{array}\right.$. So


ϖ≥λe≫θ.

Thus it follows from Proposition 2.1(v) that ϖ≫θ, i.e., ϖ∈intU. Hence, Lemma 2.2 is true.

**Lemma 2.3**  Assume the cone U⊂S is solid and β. Then α.

**Proof.**  (1) Firstly, let us show $U_{e}\subset \text{int}U.$ In fact, for any $\varpi \in U_{e}$, there exist $\lambda_{\varpi },\mu_{\varpi }>0$ such that $\lambda_{\varpi }e\leq \varpi \leq \mu_{\varpi }e$. Take $\text{Precision}=\frac{TP}{TP+FP}$. Then $\text{Recall}=\frac{TP}{TP+FN}$. So by Lemma 2.2, ϖ∈intU. Hence, $mAP=\frac{\sum_{i=1}^{N}AP_{i}}{N}$.

(2) Now we prove $\text{int}U\subset U_{e}$. For any ϖ∈intU, since e∈intU, by Lemma 2.2, there exist √ such that √ and √. Thus, √, which implies that $\varpi \in U_{e}$. Hence, $\text{int}U\subset U_{e}$.

That is to say, $U_{e}=\text{int}U.$

## 3 Fixed points for φ concave-(-ψ) convex mixed monotone operators

In this section, using some of the basic results on the abstract cones obtained in the last section, we will present a number of new fixed point theorems of ϕ concave-(−ψ) convex mixed monotone operator by virtue of cone theory as well as monotone iterative techniques in the setting of real ordered Banach spaces.

In what follows, we assume *U* is a normal cone in the real Banach space *S*.

**Theorem 3.1**  Let $\iota_{0},\;\kappa_{0}\in S$ with $\iota_{0}<\kappa_{0}$ and $ℱ:[\iota_{0},\kappa_{0}]\times [\iota_{0},\kappa_{0}]\to S$ be a ϕ concave-(−ψ) convex mixed monotone operator, where $\phi :(0,1)\times [\iota_{0},\kappa_{0}]\to (0,\infty )$ and $\psi :(0,1)\times [\iota_{0},\kappa_{0}]\to (0,\infty )$ are two functions such that $\left(t,\varpi )\in (0,1)\times [\iota_{0},\kappa_{0}\right]$ implies t<ϕ(t,ϖ)ψ(t,ϖ)≤1. Suppose that

(i) there exists $r_{0}\in {\mathbb{R}}^{+}$ such that $\iota_{0}\geq r_{0}\kappa_{0}$ and

$$\iota_{0}\leq ℱ(\iota_{0},\kappa_{0}),ℱ(\kappa_{0},\iota_{0})\leq \kappa_{0};$$ (9)

(ii) there is a point $w_{0}\in [\iota_{0},\kappa_{0}]$ such that


$$\phi (t,\varpi )\psi (t,\varpi )\geq \phi (t,w_{0})\psi (t,w_{0}),\forall (t,\varpi )\in (0,1)\times [\iota_{0},\kappa_{0}].$$

Then the operator ℱ admits the unique fixed point $\varpi^{*}$ in $\left[\iota_{0},\kappa_{0}\right]$, and for any $\left(\varpi_{0},\varrho_{0})\in [\iota_{0},\kappa_{0}]\times [\iota_{0},\kappa_{0}\right]$, the iterated sequences

$$\varpi_{m}=ℱ(\varpi_{m-1},\varrho_{m-1}),\varrho_{m}=ℱ(\varrho_{m-1},\varpi_{m-1}),m=1,2,\cdots$$ (10)

always converge to $\varpi^{*}$. Namely, $\|\varpi_{m}-\varpi^{*}\|\to 0$ and $\|\varrho_{m}-\varpi^{*}\|\to 0$ as m→∞.

**Proof.**  Now we prove the existence and the uniqueness of the fixed point. Set

$$\iota_{m}=ℱ(\iota_{m-1},\kappa_{m-1}),\;\kappa_{m}=ℱ(\kappa_{m-1},\iota_{m-1}),m=1,2,\cdots .$$ (11)

Since ℱ is mixed monotone and $\iota_{0}<\kappa_{0}$, by 3.1 we have $\iota_{0}\leq \iota_{1}\leq \kappa_{1}\leq \kappa_{0}.$ By induction, we see


$$\iota_{0}\leq \iota_{1}\leq \cdots \leq \iota_{m}\leq \cdots \leq \kappa_{m}\leq \cdots \leq \kappa_{1}\leq \kappa_{0}.$$

Obviously, $0<r_{0}\leq 1$ since $\iota_{0}\geq r_{0}\kappa_{0}$ from (i). Without loss of generality, we assume 0<*r*_0_<1. Put $t_{1}=\text{sup}\{t>0\;|\;\iota_{0}\geq t\kappa_{0}\}$, then we have $0<r_{0}\leq t_{1}\leq 1$. In general, we set

$$t_{m}=\text{sup}\{t>0\;|\;\iota_{m}\geq t\kappa_{m}\},m=1,2,\cdots .$$ (12)

Then it follows that $0\leq t_{m}\leq 1$ and

$$\iota_{m}\geq t_{m}\kappa_{m},m=1,2,\cdots .$$ (13)

By induction, we can prove that

$$0<r_{0}\leq t_{1}<t_{2}<\cdots <t_{m}<t_{m+1}<\cdots \leq 1.$$ (14)

Actually, without loss of generality, let 0<*t*_*m*_<1. From 3.5 and the fact that ℱ is ϕ concave-(−ψ) convex mixed monotone, we get

$$\iota_{m+1}=ℱ(\iota_{m},\kappa_{m})\geq ℱ(t_{m}\kappa_{m},\kappa_{m})\geq \phi (t_{m},\kappa_{m})ℱ(\kappa_{m},\kappa_{m}),$$ (15)

$$\kappa_{m+1}=ℱ(\kappa_{m},\iota_{m})\leq ℱ(\kappa_{m},t_{m}\kappa_{m})\leq \frac{1}{\psi (t_{m},\kappa_{m})}ℱ(\kappa_{m},\kappa_{m}).$$ (16)

By 3.7 and 3.8, we have

$$\iota_{m+1}\geq \phi (t_{m},\kappa_{m})\psi (t_{m},\kappa_{m})\kappa_{m+1}.$$ (17)

From 3.4 and 3.9, it follows that

$$t_{m+1}\geq \phi (t_{m},\kappa_{m})\psi (t_{m},\kappa_{m})>t_{m},m=1,2,\cdots ,$$ (18)

which implies that {*t*_*m*_} is increasing and so 3.6 holds. Hence, $\lim_{m\to \infty }t_{m}=t^{*}$ exists and $0<t^{*}\leq 1$. We now check

$$t^{*}=1.$$ (19)

Otherwise, we have $0<{\text{t}}^{*}<1$. Thus by 3.5 and the fact that ℱ is ϕ concave-(−ψ) convex mixed monotone, we see


$$\iota_{m+1}=ℱ(\iota_{m},\kappa_{m})\geq ℱ(t_{m}\kappa_{m},\kappa_{m})=ℱ(\frac{t_{m}}{t^{*}}\cdot t^{*}\kappa_{m},\kappa_{m})$$

$$\geq \phi (\frac{t_{m}}{t^{*}},t^{*}\kappa_{m})ℱ(t^{*}\kappa_{m},\kappa_{m})$$

$$\geq \phi (\frac{t_{m}}{t^{*}},t^{*}\kappa_{m})\phi (t^{*},\kappa_{m})ℱ(\kappa_{m},\kappa_{m}),$$ (20)

and


$$\kappa_{m+1}=ℱ(\kappa_{m},\iota_{m})\leq ℱ(\kappa_{m},t_{m}\kappa_{m})=ℱ(\kappa_{m},\frac{t_{m}}{t^{*}}\cdot t^{*}\kappa_{m})$$

$$\leq \frac{1}{\psi (\frac{t_{m}}{t^{*}},t^{*}\kappa_{m})}ℱ(\kappa_{m},t^{*}\kappa_{m})$$

$$\leq \frac{1}{\psi (\frac{t_{m}}{t^{*}},t^{*}\kappa_{m})}\cdot \frac{1}{\psi (t^{*},\kappa_{m})}ℱ(\kappa_{m},\kappa_{m}).$$ (21)

From (ii), 3.12 and 3.13, we get


$$\iota_{m+1}\geq \phi (\frac{t_{m}}{t^{*}},t^{*}\kappa_{m})\phi (t^{*},\kappa_{m})\psi (\frac{t_{m}}{t^{*}},t^{*}\kappa_{m})\psi (t^{*},\kappa_{m})\kappa_{m+1}$$

$$\geq \frac{t_{m}}{t^{*}}\phi (t^{*},\kappa_{m})\psi (t^{*},\kappa_{m})\kappa_{m+1}$$

$$\geq \frac{t_{m}}{t^{*}}\phi (t^{*},w_{0})\psi (t^{*},w_{0})\kappa_{m+1}.$$ (22)

It follows from 3.4 and 3.14 that

$$t_{m+1}\geq \frac{t_{m}}{t^{*}}\phi (t^{*},w_{0})\psi (t^{*},w_{0}).$$ (23)

Letting m→∞ in 3.15 we have


$$t^{*}\geq \frac{t^{*}}{t^{*}}\phi (t^{*},w_{0})\psi (t^{*},w_{0})>t^{*},$$

a contradiction. This yields t^*^ = 1. Now, for all m,j≥1, we see


$$\theta \leq \kappa_{m}-\iota_{m}\leq \kappa_{m}-t_{m}\kappa_{m}=(1-t_{m})\kappa_{m}\leq (1-t_{m})\kappa_{0}$$

and


$$\theta \leq \iota_{m+j}-\iota_{m}\leq \kappa_{m}-\iota_{m},\theta \leq \kappa_{m}-\kappa_{m+j}\leq \kappa_{m}-\iota_{m}.$$

So, on account of the normality of the cone *U*, we get $\|\kappa_{m}\;-\;\iota_{m}\|\to 0$ as m→∞ and hence $\left\{\iota_{m}\right\}$ and $\left\{\kappa_{m}\right\}$ are both Cauchy in *S*. Hence, by the fact that *S* is complete, there are two points $\iota^{*},\kappa^{*}\in S$ such that $\left\|\iota_{m}-\iota^{*}\|\to 0,\|\kappa_{m}-\kappa^{*}\|\to 0(m\to \infty \right)$ and $\iota^{*}=\kappa^{*}$. Set $\varpi^{*}=\iota^{*}=\kappa^{*}$. Since ℱ is mixed monotone, it follows from 3.2 that

$$\iota_{m}\leq \varpi_{m}\leq \kappa_{m},\iota_{m}\leq \varrho_{m}\leq \kappa_{m},m=0,1,2,\cdots .$$ (24)

Taking m→∞ in 3.16 we know $\left\|\varpi_{m}-\varpi^{*}\|\to 0,\|\varrho_{m}-\varpi^{*}\|\to 0\;(m\to \infty \right)$ and $\iota_{m}\leq \varpi^{*}\leq \kappa_{m},m=0,1,2,\cdots .$

Using standard method (see [11]) we easily show $ℱ(\varpi^{*},\varpi^{*})=\varpi^{*}$ and such fixed point $\varpi^{*}$ of ℱ is unique in $\left[\iota_{0},\kappa_{0}\right]$.

Next, we prove the convergence of the iterated sequences. For any initial $\varpi_{0},\varrho_{0}\in [\iota_{0},\kappa_{0}]$, i.e., $\iota_{0}\leq \varpi_{0}\leq \kappa_{0},\iota_{0}\leq \varrho_{0}\leq \kappa_{0},$ by 3.2 and the fact that ℱ is mixed monotone, we see


$$ℱ(\iota_{0},\kappa_{0})\leq ℱ(\varpi_{0},\varrho_{0})\leq ℱ(\kappa_{0},\iota_{0})$$

and


$$ℱ(\iota_{0},\kappa_{0})\leq ℱ(\varrho_{0},\varpi_{0})\leq ℱ(\kappa_{0},\iota_{0})$$

i.e., $\iota_{1}\leq \varpi_{1}\leq \kappa_{1}$ and $\iota_{1}\leq \varrho_{1}\leq \kappa_{1}$. It is easily seen by induction that $\iota_{m}\leq \varpi_{m}\leq \kappa_{m}$ and $\iota_{m}\leq \varrho_{m}\leq \kappa_{m},m=1,2,\ldots .$ Hence, it follows that


$$\lim_{m\to \infty }\varpi_{m}=\lim_{m\to \infty }\varrho_{m}=\varpi^{*},$$

since $\lim_{m\to \infty }\iota_{m}=\lim_{m\to \infty }\kappa_{m}=\varpi^{*}$. This shows that the convergence of the iterated sequence defined as 3.2 holds. Therefore, all the conclusions of Theorem 3.1 hold.

**Remark 3.1**  Compared with Theorem 2.1 in [11], Theorem 3.1 deletes the condition

$$\lim_{s\to t^{-}}\phi (s,w_{0})\psi (s,w_{0})>t$$ (25)

in the condition (iii) of Theorem 2.1 in [11] and the other conditions are unchangeable, but the conclusions remain hold, so Theorem 3.1 improves Theorem 2.1 in [11]. More- over, the technique in the proof of Theorem 3.1 is different from that in the proof of Theorem 2.1 in [11] since the latter depends strongly on the condition 3.17.

**Lemma 3.1**  Let ℱ:U×U→U be a ϕ concave-(−ψ) convex mixed monotone operator with ϕ(t,ϖ)≡ϕ(t) and ψ(t,ϱ)≡ψ(t) for all ϖ,ϱ∈U and t∈(0,1). Assume that there is a point e∈U with e>θ such that $ℱ(e,e)\in U_{e}$. Then $ℱ:U_{e}\;\times \;U_{e}\to U_{e}$, and moreover, there exist $\iota_{0},\;\kappa_{0}\in U_{e}$ and r∈(0,1) such that


$$r\kappa_{0}\leq \iota_{0}<\kappa_{0},\iota_{0}\leq ℱ(\iota_{0},\kappa_{0})\leq ℱ(\kappa_{0},\iota_{0})\leq \kappa_{0}.$$

**Proof.**  The method and technique to prove Lemma 3.1 are similar to that in Lemma 2.1 in [15]. We omit the proof to avoid the repeatedness.

**Theorem 3.2**  Let e>θ and $ℱ:U_{e}\times U_{e}\to U_{e}$ be a ϕ concave-(−ψ) convex mixed monotone operator with ϕ(t,ϖ)≡ϕ(t) and ψ(t,ϱ)≡ψ(t) for all $\varpi ,\varrho \in U_{e}$ and t∈(0,1). Then the operator ℱ admits the unique fixed point $\varpi^{*}$ in *U*_*e*_. Moreover, for any initial value $(\varpi_{0},\varrho_{0})\in U_{e}$, the iterated sequences


$$\varpi_{m}=ℱ(\varpi_{m-1},\varrho_{m-1}),\varrho_{m}=ℱ(\varrho_{m-1},\varpi_{m-1}),(m=1,2,\cdots )$$

always converge to $\varpi^{*}$. Namely, $\|\varpi_{m}-\varpi^{*}\|\to 0$ and $\|\varrho_{m}-\varpi^{*}\|\to 0$ as m→∞.

**Proof.**  In order to prove Theorem 3.2, we need to use Theorem 3.1. Now we will verify the operator ℱ satisfies all the conditions in Theorem 3.1.

In fact, since $e\in U_{e}$ and the operator ℱ maps $U_{e}\times U_{e}$ to *U*_*e*_, it follows that $ℱ(e,e)\in U_{e}.$ By Lemma 3.1, there exist $\iota_{0},\kappa_{0}\in U_{e}$ and r∈(0,1) such that


$$r_{0}\kappa_{0}\leq \iota_{0},\iota_{0}<\kappa_{0},\iota_{0}\leq ℱ(\iota_{0},\kappa_{0})\leq ℱ(\kappa_{0},\iota_{0})\leq \kappa_{0},$$

i.e., the condition (i) in Theorem 3.1 is satisfied.

On the other hand, since ℱ is ϕ concave-(−ψ) convex with ϕ(t,ϖ)≡ϕ(t),ψ(t,ϱ)≡ψ(t) for all $\varpi ,\varrho \in U_{e}$ and t∈(0,1), it is obvious that the condition (ii) in Theorem 3.1 is also satisfied. Hence, the conclusions of Theorem 3.2 follow from Theorem 3.1.

## 4 Fixed points for mixed monotone operators with certain concavity and convexity

In this section, we will use the main results obtained in last section to deduce some new fixed point results for mixed monotone operators with certain concavity and convexity in the setting of ordered real Banach spaces.

In what follows, *U* always represents a normal cone of the real Banach space *S*.

**Theorem 4.1**  Let e>θ and $\iota_{0},\;\kappa_{0}\in U_{e}$ with $\iota_{0}\leq \kappa_{0}$. Suppose that the operator ℱ:U×U→U is mixed monotone and satisfies the following conditions:

(i) there exists $r_{0}\in {\mathbb{R}}^{+}$ such that $\iota_{0}\geq r_{0}\kappa_{0}$ and


$$\iota_{0}\leq ℱ(\iota_{0},\kappa_{0}),ℱ(\kappa_{0},\iota_{0})\leq \kappa_{0};$$

(ii) for fixed ϱ, ℱ(·,ϱ):U→U is generalized *e*-concave (see Definition 1.4) with characteristic function η=η(t,ϖ); for fixed ϖ, ℱ(ϖ,·):U→U is (−α)-convex (see Definition 1.1). Furthermore, the number α and the function η=η(t,ϖ) satisfy

(*H*_1_) η(t,ϖ) is monotone in ϖ, and

(*H*_2_) $\left(t,\varpi )\in (0,1)\times [\iota_{0},\kappa_{0}\right]$ implies


$$\frac{1}{t^{\alpha }}-1<\eta (t,\varpi )\leq \frac{1}{t^{1+\alpha }}-1.$$

Then ℱ admits the unique fixed point $\varpi^{*}$ in $\left[\iota_{0},\kappa_{0}\right]$, and for any $\left(\varpi_{0},\varrho_{0})\in [\iota_{0},\kappa_{0}]\;\times \;[\iota_{0},\kappa_{0}\right]$, the iterated sequences


$$\varpi_{m}=ℱ(\varpi_{m-1},\varrho_{m-1}),\;\varrho_{m}=ℱ(\varrho_{m-1},\varpi_{m-1}),(m=1,2,\cdots )$$

always converge to $\varpi^{*}$. Namely, $\|\varpi_{m}-\varpi^{*}\|\to 0$ and $\|\varrho_{m}-\varpi^{*}\|\to 0$ as m→∞.

**Proof.**  According to Theorem 3.1, it suffices to check $ℱ:[\iota_{0},\kappa_{0}]\;\times \;[\iota_{0},\kappa_{0}]\to S$ is ϕ concave-(−ψ) convex with t<ϕ(t,ϖ)ψ(t,ϖ)≤1 for all $\left(t,\varpi )\in (0,1)\times [\iota_{0},\kappa_{0}\right]$, where

$$\phi (t,\varpi )=(1+\eta (t,\varpi ))t,\psi (t,\varrho )=t^{\alpha },\;\forall t\in (0,1),\;\forall \varpi ,\varrho \in [\iota_{0},\kappa_{0}].$$ (26)

Actually, since ℱ(ϖ,ϱ) is generalized *e*-concave with regard to the first variable ϖ, and is (−α)-convex with respect to the second variable ϱ, it follows that for all t∈(0,1) and $\forall \varpi ,\varrho \in [\iota_{0},\kappa_{0}]$,


ℱ(tϖ,ϱ)≥(1+η(t,ϖ))tℱ(ϖ,ϱ)=ϕ(t,ϖ)ℱ(ϖ,ϱ);

$$ℱ(\varpi ,t\varrho )\leq \frac{1}{t^{\alpha }}ℱ(\varpi ,\varrho )=\frac{1}{\psi (t,\varrho )}ℱ(\varpi ,\varrho ),$$

and moreover, we know t<ϕ(t,ϖ)ψ(t,ϖ)≤1 holds by means of (*H*_2_) and (4.1). That is to say, ℱ is ϕ concave-(−ψ) convex with ϕ and ψ satisfying 4.1. In addition, by (*H*_1_), η(t,ϖ) is monotone in ϖ, then it follows that the condition (ii) in Theorem 3.1 is satisfied. Indeed, for all $t,\varpi )\in (0,1)\times [\iota_{0},\kappa_{0}],$ we have t∈(0,1) and $\iota_{0}\leq \varpi \leq \kappa_{0},$ so it follows that

$$\eta (t,\iota_{0})\leq \eta (t,\varpi )\leq \eta (t,\kappa_{0})$$ (27)

since η(t,ϖ) is monotone in ϖ and we suppose η(t,ϖ) is nondecreasing in ϖ without loss of generality. Take $\varpi_{0}=\iota_{0}\in [\iota_{0},\kappa_{0}]$, then by 4.2 we get

$$\eta (t,\varpi )\geq \eta (t,\varpi_{0}).$$ (28)

Considering

$$\psi (t,\varpi )=\psi (t,\varpi_{0})\equiv t^{\alpha },\;\forall t\in (0,1),\;\forall \varpi \in [\iota_{0},\kappa_{0}],$$ (29)

by 4.3 and 4.4 we have


$$\phi (t,\varpi )\psi (t,\varpi )\geq \phi (t,\varpi_{0})\psi (t,\varpi_{0}),$$

which means the condition (ii) in Theorem 3.1 is satisfied. Thus, the operator ℱ meets all the conditions of Theorem 3.1. Hence, the conclusions of Theorem 4.1 hold on the basis of Theorem 3.1.

**Remark 4.1**  Compared to Theorem 2.4 in [11], Theorem 4.1 does not acquire the characteristic function η=η(t,ϖ) should satisfy the continuity condition “η(t,ϖ) is continuous in *t* from left", which is the crucial condition in (*H*_1_) of Theorem 2.4 in [11], and the conclusions stay unchanged. So Theorem 4.1 improves Theorem 2.4 in [11].

**Lemma 4.1**  Let e>θ and the operator $ℱ:U_{e}\times U_{e}\to U_{e}$. Then the following are equivalent:

(a) For any 0<*t*<1 there is 0<β=β(t)<1 such that


$$ℱ(t\varpi ,\varrho )\geq t^{\beta (t)}ℱ(\varpi ,\varrho ),\forall \varpi ,\varrho \in U_{e}.$$

(b) For any 0<*t*<1 there is 0<η=η(t)>0 such that


$$ℱ(t\varpi ,\varrho )\geq t(1+\eta (t))ℱ(\varpi ,\varrho ),\forall \varpi ,\varrho \in U_{e},$$

where t[1+η(t)]<1.

**Proof.**  In Lemma 4.1, the relation between β(t) and η(t) is such that


$$\eta (t)=t^{\beta (t)-1}-1\;\text{and}\;\beta (t)=\frac{\ln[t(1+\eta (t))]}{\ln t}.$$

**Corollary 4.1**  Let $U,S,\iota_{0},\kappa_{0}$ and ℱ be the same as in Theorem 4.1. Suppose that

(i) there exists $r_{0}\in {\mathbb{R}}^{+}$ such that $\iota_{0}\geq r_{0}\kappa_{0}$ and


$$\iota_{0}\leq ℱ(\iota_{0},\kappa_{0}),ℱ(\kappa_{0},\iota_{0})\leq \kappa_{0};$$

(ii) there exist α≥0 and a nonnegative function β=β(t) with 0<α+β(t)<1 such that for any $t\in (0,1),\varpi ,\varrho \in U_{e}$, it holds that

$$ℱ(t\varpi ,\varrho )\geq t^{\beta (t)}ℱ(\varpi ,\varrho ),$$ (30)

and

$$ℱ(\varpi ,t\varrho )\leq \frac{1}{t^{\alpha }}ℱ(\varpi ,\varrho ).$$ (31)

Then ℱ admits the unique fixed point $\varpi^{*}$ in $\left[\iota_{0},\kappa_{0}\right]$, and for any initial value $\left(\varpi_{0},\varrho_{0})\in [\iota_{0},\kappa_{0}]\times [\iota_{0},\kappa_{0}\right]$, the iterated sequences


$$\varpi_{m}=ℱ(\varpi_{m-1},\varrho_{m-1}),\varrho_{m}=ℱ(\varrho_{m-1},\varpi_{m-1}),(m=1,2,\cdots )$$

always converge to $\varpi^{*}$. Namely, $\|\varpi_{m}-\varpi^{*}\|\to 0$ and $\|\varrho_{m}-\varpi^{*}\|\to 0$ as m→∞.

**Proof.**  Let us use Theorem 4.1 to prove it. We merely need to verify that condition (ii) from Theorem 4.1 is satisfied since the condition (i) has been met in the assumption. In fact, by 4.5 and Lemma 4.1, we see

$$ℱ(t\varpi ,\varrho )\geq t(1+\eta (t))ℱ(\varpi ,\varrho ),\forall t\in (0,1),\forall \varpi ,\varrho \in U_{e},$$ (32)

where $\eta (t)=t^{\beta (t)-1}-1$. That is to say, ℱ(ϖ,ϱ) is generalized *e*-concave with its characteristic function $\eta (t)=t^{\beta (t)-1}-1$ with respect to the first variable ϖ. At the same time, 4.6 implies that ℱ(ϖ,ϱ) is (−α)-convex with regard to the second variable ϱ. In addition, the condition (*H*_1_) is satisfied since $\eta (t,\varpi )\equiv \eta (t)=t^{\beta (t)-1}-1$ for any t∈(0,1) and $\varpi \in U_{e}$. Besides, note that 0<α+β(t)≤1, then it follows that


0<1−(α+β(t))≤1,

so for any 0<t<1, we have


$$1>t^{1-(\alpha +\beta (t))}\geq t,$$

hence, we get


$$t^{\alpha }>t^{1-\beta (t)}\leq t^{1+\alpha }.$$

Then it is obviously seen that


$$\frac{1}{t^{\alpha }}<t^{\beta (t)-1}\leq \frac{1}{t^{1+\alpha }},$$

which implies that


$$\frac{1}{t^{\alpha }}-1<t^{\beta (t)-1}-1=\eta (t)<\frac{1}{t^{1+\alpha }}-1,$$

thus (*H*_2_) is also satisfied. Based on the arguments above, we know that all the conditions of Theorem 4.1 are satisfied. Thus, the conclusions hold by Theorem 4.1.

**Theorem 4.2**  Let e>θ and $\iota_{0},\;\kappa_{0}\in U_{e}$ with $\iota_{0}\leq \kappa_{0}$. Suppose that the operator ℱ:U×U→U is mixed monotone and satisfies the following conditions:

(i) $\iota_{0}\leq ℱ(\iota_{0},\kappa_{0}),ℱ(\kappa_{0},\iota_{0})\leq \kappa_{0};$

(ii) for fixed ϱ, ℱ(·,ϱ):U→U is generalized *e*-concave with characteristic function η=η(t,ϖ); for fixed ϖ, ℱ(ϖ,·):U→U is convex. Furthermore, the function η=η(t,ϖ) satisfies

(*H*_1_) η(t,ϖ) is monotone in ϖ, and

(*H*_2_) there exists ε>0 such that $ℱ(\iota_{0},\kappa_{0})\geq \varepsilon ℱ(\kappa_{0},\theta )$ and $\left(t,\varpi )\in (0,1)\times [\iota_{0},\kappa_{0}\right]$ implies

$$\frac{\left(1-t)(1-\varepsilon \right)}{\varepsilon }<\eta (t,\varpi )\leq \frac{1-t}{\varepsilon t}.$$ (33)

Then ℱ admits the unique fixed point $\varpi^{*}$ in $\left[\iota_{0},\kappa_{0}\right]$, and for any initial value $\left(\varpi_{0},\varrho_{0})\in [\iota_{0},\kappa_{0}]\times [\iota_{0},\kappa_{0}\right]$, the iterated sequences


$$\varpi_{m}=ℱ(\varpi_{m-1},\varrho_{m-1}),\varrho_{m}=ℱ(\varrho_{m-1},\varpi_{m-1}),(m=1,2,\cdots )$$

always converge to $\varpi^{*}$. Namely, $\|\varpi_{m}-\varpi^{*}\|\to 0$ and $\|\varrho_{m}-\varpi^{*}\|\to 0$ as m→∞.

**Proof.**  According to Theorem 3.1, it suffices to check $ℱ:[\iota_{0},\kappa_{0}]\times [\iota_{0},\kappa_{0}]\to S$ is ϕ concave-(−ψ) convex with t<ϕ(t,ϖ)ψ(t,ϖ)≤1 for all $\left(t,\varpi )\in (0,1)\times [\iota_{0},\kappa_{0}\right]$, where

$$\phi (t,\varpi )=(1+\eta (t,\varpi ))t,\psi (t,\varrho )=\frac{\varepsilon }{1-(1-\varepsilon )t},\;\forall t\in (0,1),\;\forall \varpi ,\varrho \in [\iota_{0},\kappa_{0}].$$ (34)

In fact, since ℱ(ϖ,ϱ) is generalized *e*-concave with respect to the first variable ϖ, and is convex with respect to the second variable ϱ, it follows from the fact the operator ℱ is mixed monotone that for all t∈(0,1) and $\varpi ,\varrho \in [\iota_{0},\kappa_{0}]$, we see


ℱ(tϖ,ϱ)≥(1+η(t,ϖ))tℱ(ϖ,ϱ)=ϕ(t,ϖ)ℱ(ϖ,ϱ),

and


ℱ(ϖ,tϱ)=ℱ(ϖ,tϱ+(1−t)θ)≤tℱ(ϖ,ϱ)+(1−t)ℱ(ϱ,θ)

$$\leq tℱ(\varpi ,\varrho )+(1-t)ℱ(\kappa_{0},\theta )\leq tℱ(\varpi ,\varrho )+\frac{1-t}{\varepsilon }ℱ(\iota_{0},\kappa_{0})$$

$$\leq tℱ(\varpi ,\varrho )+\frac{1-t}{\varepsilon }ℱ(\varpi ,\varrho )={\left[\frac{\varepsilon }{\varepsilon t+(1-t)}\right]}^{-1}ℱ(\varpi ,\varrho )=\psi (t,\varrho )ℱ(\varpi ,\varrho ).$$

Moreover, we know t<ϕ(t,ϖ)ψ(t,ϖ)≤1 holds by virtue of 4.8 and 4.9. That is to say, ℱ is ϕ concave-(−ψ) convex. In addition, by (*H*_1_), η(t,ϖ) is monotone in ϖ, then it follows that the condition (ii) in Theorem 3.1 is satisfied. In fact, for all $t,\varpi )\in (0,1)\times [\iota_{0},\kappa_{0}],$ we see t∈(0,1) and $\iota_{0}\leq \varpi \leq \kappa_{0}.$

Take $\varpi_{0}=\iota_{0}\in [\iota_{0},\kappa_{0}].$ Since η(t,ϖ) is monotone in ϖ, without loss of generality, suppose η(t,ϖ) is nondecreasing in ϖ, then we have


$$\eta (t,\varpi_{0})\leq \eta (t,\varpi )\leq \eta (t,\kappa_{0}),$$

so it follows that


$$(1+\eta (t,\varpi ))t\geq (1+\eta (t,\varpi_{0}))t,$$

i.e., $\phi (t,\varpi )\geq \phi (t,\varpi_{0}).$ Considering


$$\psi (t,\varpi )\equiv \psi (t,\varpi_{0})=\frac{1}{1-(1-\varepsilon )t},\;\forall t\in (0,1),\;\forall \varpi \in [\iota_{0},\kappa_{0}],$$

we have


$$\phi (t,\varpi )\psi (t,\varpi_{0})\geq \phi (t,\varpi_{0})\psi (t,\varpi_{0}),$$

which yields the condition (ii) in Theorem 3.1 is satisfied. Thus all the conditions of Theorem 3.1 are met for the operator ℱ. Hence, the conclusions of Theorem 4.2 hold on the basis of Theorem 3.1.

**Remark 4.2**  Compared to Theorem 2.5 in [11], Theorem 4.2 does not require the characteristic function η=η(t,ϖ) to satisfy the continuity condition “η(t,ϖ) is continuous in *t* from left", which is the crucial condition in (*H*_1_) of Theorem 2.5 in [11], and the conclusions remain the same. So Theorem 4.2 improves Theorem 2.5 in [11].

**Theorem 4.3**  Let e>θ and $\iota_{0},\;\kappa_{0}\in U_{e}$ with $\iota_{0}\leq \kappa_{0}$. Suppose that the operator ℱ:U×U→U is mixed monotone and satisfies the following conditions:

(i) there exists $r_{0}\in {\mathbb{R}}^{+}$ such that $\iota_{0}\geq r_{0}\kappa_{0}$;

(ii) $\iota_{0}\leq ℱ(\iota_{0},\kappa_{0}),ℱ(\kappa_{0},\iota_{0})\leq \kappa_{0};$

(iii) for fixed ϱ, ℱ(·,ϱ):U→U is generalized *e*-concave with characteristic function η=η(t,ϖ); for fixed ϖ, ℱ(ϖ,·):U→U is generalized *e*-convex with characteristic function ξ=ξ(t,ϱ). Furthermore, these functions η=η(t,ϖ) and ξ=ξ(t,ϖ) satisfy the following

(*H*_1_) the function (1+η(t,ϖ))(1+ξ(t,ϖ)) is monotone in ϖ, and

(*H*_2_) for all $\left(t,\varpi )\in (0,1)\times [\iota_{0},\kappa_{0}\right]$, it holds that

$$\frac{1-t}{t}<\eta +\xi +\eta \xi \leq \frac{1-t^{2}}{t^{2}}.$$ (35)

Then ℱ admits the unique fixed point $\varpi^{*}$ in $\left[\iota_{0},\kappa_{0}\right]$, and for any $\left(\varpi_{0},\varrho_{0})\in [\iota_{0},\kappa_{0}]\;\times \;[\iota_{0},\kappa_{0}\right]$, the iterated sequences


$$\varpi_{m}=ℱ(\varpi_{m-1},\varrho_{m-1}),\varrho_{m}=ℱ(\varrho_{m-1},\varpi_{m-1}),(m=1,2,\cdots )$$

always converge to $\varpi^{*}$. Namely, $\|\varpi_{m}-\varpi^{*}\|\to 0$ and $\|\varrho_{m}-\varpi^{*}\|\to 0$ as m→∞.

**Proof.**  According to Theorem 3.1, it suffices to check that $ℱ:[\iota_{0},\kappa_{0}]\times [\iota_{0},\kappa_{0}]\to S$ is ϕ concave-(−ψ) convex, where


$$\phi (t,\varpi )=t(1+\eta (t,\varpi )),\psi (t,\varrho )=t(1+\xi (t,\varrho )),\forall t\in (0,1),\varpi ,\varrho \in [\iota_{0},\kappa_{0}].$$

In fact, for all t∈(0,1) and $\varpi ,\varrho \in [\iota_{0},\kappa_{0}]$, we have


ℱ(tϖ,ϱ)≥t(1+η(t,ϖ))ℱ(ϖ,ϱ)=ϕ(t,ϖ)ℱ(ϖ,ϱ),

$$ℱ(\varpi ,t\varrho )\leq \frac{1}{t(1+\xi (t,\varrho ))}ℱ(\varpi ,\varrho )=\frac{1}{\psi (t,\varrho )}ℱ(\varpi ,\varrho )$$

and moreover, t<ϕ(t,ϖ)ψ(t,ϖ)≤1 holds by virtue of (4.10). So, $ℱ:[\iota_{0},\kappa_{0}]\times [\iota_{0},\kappa_{0}]\to S$ is ϕ concave-(−ψ) convex. Hence the conclusions of Theorem 4.3 follow from Theorem 3.1.

**Theorem 4.4**  Let e>θ and $\iota_{0},\;\kappa_{0}\in U_{e}$ with $\iota_{0}<\kappa_{0}$. Suppose that the operator ℱ:U×U→U is mixed monotone and satisfies the following conditions:

$$\text{(i)}\;\iota_{0}\leq ℱ(\iota_{0},\kappa_{0}),ℱ(\kappa_{0},\iota_{0})\leq \kappa_{0};$$ (36)

(ii) for fixed ϱ, ℱ(·,ϱ):U→U is concave; for fixed ϖ, ℱ(ϖ,·):U→U is generalized *e*-convex with characteristic function ξ=ξ(t,ϱ). Moreover, the function ξ=ξ(t,ϱ) satisfies

(*H*_1_) ξ(t,ϱ)) is monotone in ϱ, and

(*H*_2_) there exists ε>0 such that $ℱ(\theta ,\kappa_{0})\geq \varepsilon ℱ(\kappa_{0},\iota_{0})$ and $\left(t,\varpi )\in (0,1)\times [\iota_{0},\kappa_{0}\right]$ implies

$$\frac{1}{\varepsilon +(1-\varepsilon )t}-1<\xi (t,\varpi )\leq \frac{1}{\varepsilon t+(1-\varepsilon )t^{2}}-1.$$ (37)

Then ℱ admits the unique fixed point $\varpi^{*}$ in $\left[\iota_{0},\kappa_{0}\right]$, and for any initial value $\left(\varpi_{0},\varrho_{0})\in [\iota_{0},\kappa_{0}]\times [\iota_{0},\kappa_{0}\right]$, the iterated sequences


$$\varpi_{m}=ℱ(\varpi_{m-1},\varrho_{m-1}),\varrho_{m}=ℱ(\varrho_{m-1},\varpi_{m-1}),(m=1,2,\cdots )$$

always converge to $\varpi^{*}$. Namely, $\|\varpi_{m}-\varpi^{*}\|\to 0$ and $\|\varrho_{m}-\varpi^{*}\|\to 0$ as m→∞.

**Proof.**  Let


$$\iota_{m}=ℱ(\iota_{m-1},\kappa_{m-1}),\;\kappa_{m}=ℱ(\kappa_{m-1},\iota_{m-1}),m=1,2,\cdots .$$

By 4.11 and the fact that ℱ is mixed monotone, we have

$$\iota_{0}\leq \iota_{1}\leq \cdots \leq \iota_{m}\leq \cdots \leq \kappa_{m}\leq \cdots \leq \kappa_{1}\leq \kappa_{0}.$$ (38)

Since ℱ is mixed monotone, by (*H*_2_), it follows that $\iota_{1}\geq \varepsilon \kappa_{1}$, thus 0<ε≤1. Now, we begin to show $ℱ:[\iota_{1},\kappa_{1}]\times [\iota_{1},\kappa_{1}]\to S$ is ϕ concave-(−ψ) convex. It is sufficient to prove $ℱ:[\iota_{0},\kappa_{0}]\times [\iota_{0},\kappa_{0}]\to S$ is ϕ concave-(−ψ) convex, where

$$\phi (t,\varpi )=t+\varepsilon (1-t),\psi (t,\varrho )=t(1+\xi (t,\varrho )),\forall t\in (0,1),\forall \varpi ,\varrho \in [\iota_{0},\kappa_{0}].$$ (39)

In fact, for all $\left(t,\varpi ,\varrho )\in (0,1)\times [\iota_{0},\kappa_{0}]\times [\iota_{0},\kappa_{0}\right]$, we have


ℱ(tϖ,ϱ)=ℱ(tϖ+(1−t)θ,ϱ)≥tℱ(ϖ,ϱ)+(1−t)ℱ(θ,ϱ)

$$\geq tℱ(\varpi ,\varrho )+(1-t)ℱ(\theta ,\kappa_{0})\geq tℱ(\varpi ,\varrho )+\varepsilon (1-t)ℱ(\kappa_{0},\iota_{0})$$

≥tℱ(ϖ,ϱ)+ε(1−t)ℱ(ϖ,ϱ)=ϕ(t,ϖ)ℱ(ϖ,ϱ),

$$ℱ(\varpi ,t\varrho )\leq \frac{1}{t(1+\xi (t,\varrho ))}ℱ(\varpi ,\varrho )=\frac{1}{\psi (t,\varrho )}ℱ(\varpi ,\varrho ).$$

By 4.12 and 4.14, we obtain t<ϕ(t,ϖ)ψ(t,ϖ)≤1 holds for all $\left(t,\varpi )\in (0,1)\times [\iota_{0},\kappa_{0}\right]$. Hence, based on the arguments above we see $ℱ:[\iota_{1},\kappa_{1}]\;\times \;[\iota_{1},\kappa_{1}]\to S$ is ϕ concave-(−ψ) convex, and so all the conditions of Theorem 3.1 are satisfied, which means that Theorem 4.4 holds on the basis of Theorem 3.1.

**Remark 4.3**  Compared to Corollary 3.3 in [20], Theorem 4.4 deletes the continuity condition “ξ(t,ϱ) is continuous in *t* from left", which is the crucial condition the characteristic function ξ=ξ(t,ϱ) should satisfy in the assumption (iii) of Corollary 3.3 in [20], and the conclusions remain the same. So Theorem 4.4 improves [20], Corollary 3.3].

**Theorem 4.5**  Let e>θ and $\iota_{0},\;\kappa_{0}\in U_{e}$ with $\iota_{0}\leq \kappa_{0}$. Suppose that the operator ℱ:U×U→U is mixed monotone and satisfies the following conditions:

(i) there exists $r_{0}\in {\mathbb{R}}^{+}$ such that $\iota_{0}\geq r_{0}\kappa_{0}$;

(ii) $\iota_{0}\leq ℱ(\iota_{0},\kappa_{0}),ℱ(\kappa_{0},\iota_{0})\leq \kappa_{0};$

(iii) for fixed ϱ, ℱ(·,ϱ):U→U is α-concave; for fixed ϖ, ℱ(ϖ,·):U→U is generalized *e*-convex with characteristic function ξ=ξ(t,ϱ). Moreover, the function ξ=ξ(t,ϱ) satisfies

(*H*_1_) the function ξ(t,ϱ) is monotone in ϱ, and

(*H*_2_) for all $\left(t,\varpi )\in (0,1)\times [\iota_{0},\kappa_{0}\right]$, it holds that

$$\frac{1}{t^{\alpha }}-1<\xi (t,\varpi )\leq \frac{1}{t^{1+\alpha }}-1.$$ (40)

Then ℱ admits the unique fixed point $\varpi^{*}$ in $\left[\iota_{0},\kappa_{0}\right]$, and for any $\left(\varpi_{0},\varrho_{0})\in [\iota_{0},\kappa_{0}]\;\times \;[\iota_{0},\kappa_{0}\right]$, the iterated sequences


$$\varpi_{m}=ℱ(\varpi_{m-1},\varrho_{m-1}),\varrho_{m}=ℱ(\varrho_{m-1},\varpi_{m-1}),(m=1,2,\cdots )$$

always converge to $\varpi^{*}$. Namely, $\|\varpi_{m}-\varpi^{*}\|\to 0$ and $\|\varrho_{m}-\varpi^{*}\|\to 0$ as m→∞.

**Proof.**  According to Theorem 3.1, it suffices to check that $ℱ:[\iota_{0},\kappa_{0}]\times [\iota_{0},\kappa_{0}]\to S$ is ϕ concave-(−ψ) convex, where


$$\phi (t,\varpi )=t^{\alpha },\psi (t,\varrho )=t(1+\xi (t,\varrho )),\forall t\in (0,1),\varpi ,\varrho \in [\iota_{0},\kappa_{0}].$$

In fact, for all t∈(0,1) and $\varpi ,\varrho \in [\iota_{0},\kappa_{0}]$, we have


$$ℱ(t\varpi ,\varrho )\geq t^{\alpha }ℱ(\varpi ,\varrho )=\phi (t,\varpi )ℱ(\varpi ,\varrho ),$$

$$ℱ(\varpi ,t\varrho )\leq \frac{1}{t(1+\xi (t,\varrho ))}ℱ(\varpi ,\varrho )=\frac{1}{\psi (t,\varrho )}ℱ(\varpi ,\varrho )$$

and moreover, t<ϕ(t,ϖ)ψ(t,ϖ)≤1 holds by virtue of 4.15. Hence, $ℱ:[\iota_{0},\kappa_{0}]\times [\iota_{0},\kappa_{0}]\to S$ is ϕ concave-(−ψ) convex. The conclusions of Theorem 4.5 hold by virtue of Theorem 3.1.

**Theorem 4.6**  Let e>θ and $ℱ:U_{e}\times U_{e}\to U_{e}$ be a mixed monotone operator. If there exist $\alpha_{1},\alpha_{2}\in [0,1)$ such that $0\leq \alpha_{1}+\alpha_{2}<1$ and

(H) for fixed ϱ, $ℱ(\cdot ,\varrho ):U_{e}\to U_{e}$ is $\alpha_{1}$-concave; for fixed ϖ, $ℱ(\varpi ,\cdot ):U_{e}\to U_{e}$ is $\left(-\alpha_{2}\right)$-convex, then the operator ℱ admits the unique fixed point $\varpi^{*}$ in *U*_*e*_. Moreover, for any initial value $(\varpi_{0},\varrho_{0})\in U_{e}$, the iterated sequences


$$\varpi_{m}=ℱ(\varpi_{m-1},\varrho_{m-1}),\varrho_{m}=ℱ(\varrho_{m-1},\varpi_{m-1}),(m=1,2,\cdots )$$

always converge to $\varpi^{*}$. Namely, $\|\varpi_{m}-\varpi^{*}\|\to 0$ and $\|\varrho_{m}-\varpi^{*}\|\to 0$ as m→∞.

**Proof.**  Obviously, it can be shown that the operator ℱ is ϕ concave-(−ψ) convex mixed monotone, where


$$\phi (t,\varpi )\equiv \phi (t)=t^{\alpha_{1}},\psi (t,\varrho )\equiv \psi (t)=t^{\alpha_{2}},\forall \varpi ,\varrho \in U_{e}.$$

Note that for all $t\in (0,1),\varpi ,\varrho \in U_{e}$, since $0\leq \alpha_{1}+\alpha_{2}<1$, it follows that


$$1\geq t^{\alpha_{1}+\alpha_{2}}>t.$$

Namely, $t<\phi (t,\varpi )\psi (t,\varpi )\leq 1,\forall (t,\varpi )\in (0,1)\;\times \;U_{e}.$ So, ℱ is ϕ concave-(−ψ) convex mixed monotone operator with ϕ(t,ϖ)≡ϕ(t) and ψ(t,ϱ)≡ψ(t) indeed. Hence, the conclusions of Theorem 4.6 hold by virtue of Theorem 3.2.

**Corollary 4.2**  Let *U* be solid and ℱ:intU×intU→intU be a mixed monotone operator. If there exist $\alpha_{1},\alpha_{2}\in [0,1)$ such that $0\leq \alpha_{1}+\alpha_{2}<1$ and

(H) for fixed ϱ, ℱ(·,ϱ):intU→intU is $\alpha_{1}$-concave; for fixed ϖ, ℱ(ϖ,·):intU→intU is $\left(-\alpha_{2}\right)$-convex,

then the operator ℱ admits the unique fixed point $\varpi^{*}$ in intU. Moreover, for any initial value $(\varpi_{0},\varrho_{0})\in \text{int}U$, the iterated sequences


$$\varpi_{m}=ℱ(\varpi_{m-1},\varrho_{m-1}),\varrho_{m}=ℱ(\varrho_{m-1},\varpi_{m-1}),(m=1,2,\cdots )$$

always converge to $\varpi^{*}$. Namely, $\|\varpi_{m}-\varpi^{*}\|\to 0$ and $\|\varrho_{m}-\varpi^{*}\|\to 0$ as m→∞.

**Proof.**  Since *U* is solid, intU≠∅. Hence there exists e∈intU. By Lemma 2.3, we have $U_{e}=\text{int}U$, which finishes the proof of Corollary 4.2 by Theorem 4.6.

Obviously, the following simple result is derived from Corollary 4.2; the proof is omitted.

**Corollary 4.3**  Let U,S,ℱ be the same as in Corollary 4.2. Assume that there exists $\alpha \in [0,\frac{1}{2})$ such that

(H) for fixed ϱ, ℱ(·,ϱ):intU→intU is α-concave; for fixed ϖ, ℱ(ϖ,·):intU→intU is (−α)-convex.

Then the operator ℱ admits the unique fixed point $\varpi^{*}$ in intU. Moreover, for any $(\varpi_{0},\varrho_{0})\in \text{int}U$, the iterated sequences


$$\varpi_{m}=ℱ(\varpi_{m-1},\varrho_{m-1}),\varrho_{m}=ℱ(\varrho_{m-1},\varpi_{m-1}),(m=1,2,\cdots )$$

always converge to $\varpi^{*}$. Namely, $\|\varpi_{m}-\varpi^{*}\|\to 0$ and $\|\varrho_{m}-\varpi^{*}\|\to 0$ as m→∞.

**Remark 4.4**  Compared with Corollary 2.1 and Corollary 2.2 in [11], Corollary 4.2 and Corollary 4.3 delete the following condition of coupled upper and lower solutions

(ii) there exist two points $\iota_{0},\;\kappa_{0}\in \text{int}U$ with $\iota_{0}\leq \kappa_{0}$ such that


$$\iota_{0}\leq ℱ(\iota_{0},\kappa_{0}),ℱ(\kappa_{0},\iota_{0})\leq \kappa_{0}$$

in Corollary 2.1 and Corollary 2.2 in [11], and the other conditions remain unchangeable, while the conclusions are the same. So Corollary 4.2 and Corollary 4.3 improve Corollary 2.1 and Corollary 2.2 in [11], respectively.

## 5 Applications

In this section, we will give applications of some main results gained above to nonlinear integral equations. As a result, it shows that the fixed point theorems of ϕ concave-(−ψ) convex mixed monotone operator obtained in the previous section are powerful to study the existence and uniqueness of the positive solutions to two classes of nonlinear integral equations.

**Theorem 5.1**  Let *G* be a closed subset of ${\mathbb{R}}^{N}$. Suppose K:G×G→(0,∞) is a continuous function and α,β are two positive real numbers with 0<α+β<1. Then the following nonlinear nonlinear integral equation

$$\varpi (t)=(ℱ\varpi )(t)=\int_{G}K(t,s)[\varpi^{\alpha }(s)+\varpi^{-\beta }(s)]ds$$ (41)

admits a unique positive solution $\varpi^{*}(t)$. Moreover, the iterated sequences $\{\varpi_{m}(t){\}}_{m=1}^{\infty }$ and $\{\varrho_{m}(t){\}}_{m=1}^{\infty }$ with


$$\varpi_{m}(t)=\int_{G}K(t,s)[\varpi_{m-1}^{\alpha }(s)+\varrho_{m-1}^{-\beta }(s)]ds$$

and


$$\varrho_{m}(t)=\int_{G}K(t,s)[\varrho_{m-1}^{\alpha }(s)+\varpi_{m-1}^{-\beta }(s)]ds$$

for any initial $\left(\varpi_{0},\varrho_{0})\in (0,\infty )\times (0,\infty \right)$, we have


$$\sup_{t\in G}|\varpi_{m}(t)-\varpi^{*}(t)|\to 0,\sup_{t\in G}|\varrho_{m}(t)-\varpi^{*}(t)|\to 0$$

as m→∞.

**Proof.**  Let S=C(G), the space of continuous bounded functions on *G*. We define $\left\|\varpi \|=\underset{t\in G}{\sup}|\varpi (t)\right|$, then *S* is a Banach space. Let


U={ϖ(t)∈C(G)|ϖ(t)≥0,∀t∈G}.

It is not difficult to observe that *U* is a normal solid cone in *S* and intU={ϖ(t)∈C(G)|ϖ(t)>0,∀t∈G}. Obviously, can be written as ϖ=ℱ(ϖ,ϖ), where


$$ℱ(\varpi ,\varrho )={ℱ}_{1}(\varpi )+{ℱ}_{2}(\varrho ),$$

$${ℱ}_{1}(\varpi )=\int_{G}K(t,s)\varpi^{\alpha }(s)ds,{ℱ}_{2}(\varrho )=\int_{G}K(t,s)\varrho^{-\beta }(s)ds.$$

We now confirm that the operator ℱ fulfills all the requirements specified in Corollary 4.2. In fact, put $\alpha_{1}=\alpha$ and $\alpha_{2}=\beta$, then ℱ:intU×intU→intU is a mixed monotone operator and for fixed ϱ, ℱ(·,ϱ):intU→intU is $\alpha_{1}$-concave; for fixed ϖ, ℱ(ϖ,·):intU→intU is $\left(-\alpha_{2}\right)$-convex, where $0<\alpha_{1}+\alpha_{2}=\alpha +\beta <1$. That is to say, all the conditions in Corollary 4.2 are satisfied. As a results, the conclusions of Theorem 5.1 hold by Corollary 4.2.

**Remark 5.1**  If one takes $G={\mathbb{R}}^{N},\alpha =\frac{1}{2},\beta =\frac{1}{3}$ in Theorem 5.1, then Theorem 5.1 is reduced to the case of [11, Example 3.1]. Obviously, Theorem 5.1 extends and improves [11, Example 3.1], since Theorem 5.1 concludes that has a unique positive solution in intU, the set of all positive continuous bounded functions on *G*, while Example 3.1 in [11] can only state that has a unique positive solution in a given interval such as [10^−2^,1], but cannot in the whole intU.

In what follows, we will use $S=C_{B}({\mathbb{R}}^{N})$ to represent the family of all bounded continuous functions of ${\mathbb{R}}^{N}$. Define the norm $\left\|\varpi \|=\sup_{t\in {\mathbb{R}}^{N}}|\varpi (t)\right|$. Then *S* is a real Banach space. Denote by $U=C_{B}^{+}({\mathbb{R}}^{N})$ all the nonnegative functions in $C_{B}({\mathbb{R}}^{N})$. It is obvious that *S* is ordered by the normal cone *U* with the order relation as ϖ≤ϱ if and only if $\varpi (t)\leq \varrho (t)\;(t\in {\mathbb{R}}^{N})$ for all ϖ=ϖ(t),ϱ=ϱ(t)∈S.

**Theorem 5.2**  Let $K:{\mathbb{R}}^{N}\times {\mathbb{R}}^{N}\to {\mathbb{R}}^{1}$ be a nonnegative and continuous function. Consider the following Hammerstein integral equation:

$$\varpi (t)=(ℱ\varpi )(t)=\int_{{\mathbb{R}}^{N}}K(t,s)[f(\varpi (s))+\varpi^{-\alpha }(s)]ds,$$ (42)

where 0<α<1 is a real constant and $f:C_{B}^{+}({\mathbb{R}}^{N})\to C_{B}^{+}({\mathbb{R}}^{N}).$ Further, suppose the following conditions are satisfied:

(i) *f* is nondecreasing, i.e., $\forall \varpi_{1}(t),\varpi_{2}(t)\in C_{B}^{+}({\mathbb{R}}^{N}),$


$$\varpi_{1}(t)\leq \varpi_{2}(t)\;(\forall t\in {\mathbb{R}}^{N})\Rightarrow f(\varpi_{1}(t))\leq f(\varpi_{2}(t));$$

(ii) for any $\lambda \in (0,1),\varpi \in C_{B}^{+}({\mathbb{R}}^{N})$, there exists a real function β=β(λ) with β(λ)>0 and 0<α+β(λ)<1 such that


$$f(\lambda \varpi )\geq \lambda^{\beta (\lambda )}f(\varpi );$$

(iii) there exist two functions $\iota_{0}=\iota_{0}(t),\kappa_{0}=\kappa_{0}(t)\in C_{B}^{+}({\mathbb{R}}^{N})$ and a real number *r*_0_>0 with $\iota_{0}(t)\geq r_{0}\kappa_{0}(t)\;(\forall t\in {\mathbb{R}}^{N})$ such that


$$\iota_{0}(t)\leq \int_{{\mathbb{R}}^{N}}K(t,s)[f(\iota_{0}(s))+\kappa_{0}^{-\alpha }(s)]ds$$

$$\kappa_{0}(t)\geq \int_{{\mathbb{R}}^{N}}K(t,s)[f(\kappa_{0}(s))+\iota_{0}^{-\alpha }(s)]ds.$$

Then admits a unique continuous positive solution $\varpi^{*}(t)$ with $\iota_{0}(t)\leq \varpi^{*}(t)\leq$ $\kappa_{0}(t)$ $\left(\forall t\in {\mathbb{R}}^{N}\right)$. Furthermore, for any initial value $\left(\varpi_{0},\varrho_{0})\in C_{B}^{+}({\mathbb{R}}^{N})\times C_{B}^{+}({\mathbb{R}}^{N}\right)$ with $\iota_{0}(t)\leq \varpi_{0}(t)\leq \kappa_{0}(t)$ and $\iota_{0}(t)\leq \varrho_{0}(t)\leq \kappa_{0}(t)$ $\left(\forall t\in {\mathbb{R}}^{N}\right)$, the sequences $\left\{\varpi_{m}(t)\right\}$ and $\left\{\varrho_{m}(t)\right\}$ with


$$\varpi_{m}(t)=\int_{{\mathbb{R}}^{N}}K(t,s)[f(\varpi_{m-1}(s))+\varrho_{m-1}^{-\alpha }(s)]ds$$

and


$$\varrho_{m}(t)=\int_{{\mathbb{R}}^{N}}K(t,s)[f(\varrho_{m-1}(s))+\varpi_{m-1}^{-\alpha }(s)]ds$$

always converge to $\varpi^{*}(t)$. Namely,


$$\sup_{t\in {\mathbb{R}}^{N}}|\varpi_{m}(t)-\varpi^{*}(t)|\to 0\;\text{and}\;\sup_{t\in {\mathbb{R}}^{N}}|\varrho_{m}(t)-\varpi^{*}(t)|\to 0\;\text{as}\;m\to \infty .$$

**Proof.**  It is easily seen that Eq. (5.2) can be written as ϖ=ℱ(ϖ,ϖ), where $ℱ(\varpi ,\varrho )={ℱ}_{1}(\varpi )+{ℱ}_{2}(\varrho ).$ Here ${ℱ}_{1}(\varpi )$ and ${ℱ}_{2}(\varrho )$ are defined as

$${ℱ}_{1}(\varpi )=\int_{{\mathbb{R}}^{N}}K(t,s)f(\varpi (s))ds,\;{ℱ}_{2}(\varrho )=\int_{{\mathbb{R}}^{N}}K(t,s)\varrho^{-\alpha }(s)ds.$$ (43)

We will prove Theorem 5.2 by means of Corollary 4.1. It is sufficient to check that the operator ℱ satisfies all the conditions of Corollary 4.1.

At first, we can demonstrate that ℱ(ϖ,ϱ) is a mixed monotone operator. Actually, for any $\varpi_{i},\varrho_{i}\in U=C_{B}^{+}({\mathbb{R}}^{N})(i=1,2)$, suppose that $\varpi_{i}=\varpi_{i}(t),\varrho_{i}=\varrho_{i}(t)$ with $\varpi_{1}\leq \varpi_{2},\varrho_{2}\leq \varrho_{1}$ (that is, $\varpi_{1}(t)\leq \varpi_{2}(t),\varrho_{2}(t)\leq \varrho_{1}(t),\forall t\in {\mathbb{R}}^{N})$, then by (i) and 0<α<1 we see


$$ℱ(\varpi_{1},\varrho_{1})=\int_{{\mathbb{R}}^{N}}K(t,s)[f(\varpi_{1}(s))+\varrho_{1}^{-\alpha }(s)]ds$$

$$\leq \int_{{\mathbb{R}}^{N}}K(t,s)[f(\varpi_{2}(s))+\varrho_{2}^{-\alpha }(s)]ds$$

$$=ℱ(\varpi_{2},\varrho_{2}),$$

which means ℱ:U×U→U is mixed monotone.

Next, by (iii) and 5.3, we see that there exist $\iota_{0},\kappa_{0}\in U$ and *r*_0_>0 with $\iota_{0}\geq r_{0}\kappa_{0}$ such that


$$\iota_{0}\leq ℱ(\iota_{0},\kappa_{0}),ℱ(\kappa_{0},\iota_{0})\leq \kappa_{0}$$

so the condition (i) in Corollary 4.1 is satisfied.

Thirdly, let us check the condition (ii) in Corollary 4.1 is also satisfied. In fact, by (ii), for any $\lambda \in (0,1),\varpi \in C_{B}^{+}({\mathbb{R}}^{N})=U$, there exists β=β(λ) with β(λ)>0 and 0<α+β(λ)<1 such that


$$f(\lambda \varpi )\geq \lambda^{\beta (\lambda )}f(\varpi ),$$

so it follows that for given ϱ∈U and for any λ∈(0,1) and ϖ∈U, we have


$$ℱ(\lambda \varpi ,\varrho )={ℱ}_{1}(\lambda \varpi )+{ℱ}_{2}(\varrho )$$

$$=\int_{{\mathbb{R}}^{N}}K(t,s)f(\lambda \varpi (s))ds+\int_{{\mathbb{R}}^{N}}K(t,s)\varrho^{-\alpha }(s)ds$$

$$\geq \lambda^{\beta (\lambda )}\int_{{\mathbb{R}}^{N}}K(t,s)f(\varpi (s))ds+\int_{{\mathbb{R}}^{N}}K(t,s)\varrho^{-\alpha }(s)ds$$

$$\geq \lambda^{\beta (\lambda )}\int_{{\mathbb{R}}^{N}}K(t,s)\left[f(\varpi (s))ds+\varrho^{-\alpha }(s)\right]ds$$

$$=\lambda^{\beta (\lambda )}ℱ(\varpi ,\varrho ),$$ (44)

noting that in 5.4 since λ∈(0,1) and β(λ)>0 we see $0<\lambda^{\beta (\lambda )}<1$.

On the other hand, for given ϖ∈U and for any λ∈(0,1) and ϱ∈U, we get


$$ℱ(\varpi ,\lambda \varrho )={ℱ}_{1}(\varpi )+{ℱ}_{2}(\lambda \varrho )$$

$$=\int_{{\mathbb{R}}^{N}}K(t,s)f(\varpi (s))ds+\int_{{\mathbb{R}}^{N}}K(t,s)[\lambda \varrho (s){]}^{-\alpha }ds$$

$$=\int_{{\mathbb{R}}^{N}}K(t,s)f(\varpi (s))ds+\lambda^{-\alpha }\int_{{\mathbb{R}}^{N}}K(t,s)\varrho^{-\alpha }(s)ds$$

$$\leq \lambda^{-\alpha }\left[\int_{{\mathbb{R}}^{N}}K(t,s)f(\varpi (s))ds+\int_{{\mathbb{R}}^{N}}K(t,s)\varrho^{-\alpha }(s)ds\right]$$

$$=\lambda^{-\alpha }ℱ(\varpi ,\varrho ),$$ (45)

noting that in 5.5 since 0<λ<1 and 0<α<1 we see $1>\lambda^{\alpha }>\lambda$ and so $1<\lambda^{-\alpha }$.

By 5.4 and 5.5 we hold that the condition (ii) in Corollary 4.1 is satisfied. In a word, all the conditions of Corollary 4.1 are satisfied. Therefore, the conclusions of Theorem 5.2 hold by Corollary 4.1.

**Remark 5.2**  In Corollary 4.1, if we take β(t)≡β as a constant with 0<β<1 and 0<α+β<1, then Corollary 4.1 is reduced to [11], Theorem 2.6]. So Corollary 4.1 generalizes [11], Theorem 2.6] and also Theorem 5.2 generalizes [11], Example 3.1].

## 6 Conclusions

In this paper, we prove some useful results about abstract cone and partial order in real ordered Banach spaces. Utilizing these results, we obtain a number of new fixed point theorems of ϕ concave-(−ψ) convex mixed monotone operators by means of monotone iterative techniques in real ordered Banach spaces with the normal cones. Then, these results continue to deduce other new fixed point results for mixed monotone operators with certain concavity and convexity. Our main results improve and extend the previous findings. In order to explain these results, we show that the results are powerful to study the existence and uniqueness of the positive solutions to two classes of nonlinear integral equations. The main novelty of this paper is that much deeper fixed point results about mixed monotone operators with certain concavity and convexity are presented by virtue of a number of new monotone iterative techniques in the setting of ordered Banach spaces. Future research will be focused on the applications of the obtained new fixed point results regarding mixed monotone operators to nonlinear mixed monotone systems and discuss their global dynamical properties.
